# Effect of tanniniferous food from *Bauhinia pulchella* on pasture contamination with gastrointestinal nematodes from goats

**DOI:** 10.1186/s13071-016-1370-3

**Published:** 2016-02-24

**Authors:** Suzana G. Lopes, Lilyan B. G. Barros, Helder Louvandini, Adibe L. Abdalla, Livio M. Costa Junior

**Affiliations:** Curso de Licenciatura em Educação do Campo-Ciências da Natureza, Universidade Federal do Piauí, Picos, Piauí Brazil; Laboratório de Controle de Parasitos, Centro de Ciências Biológicas e da Saúde, Departamento de Patologia, Universidade Federal do Maranhão, São Luis, Maranhão Brazil; Laboratório de Nutrição Animal, Centro de Energia Nuclear na Agricultura, Universidade de São Paulo, Piracicaba, São Paulo Brazil

**Keywords:** Tannin, Small ruminant, Livestock, Anthelmintic

## Abstract

**Background:**

Tannin-rich plants have been examined as an alternative for controlling the gastrointestinal nematodes in ruminants. *In vivo* assays typically examine the anthelmintic activity in female fecundity and/or the adult worm burden, without considering other life-cycle stages or the impact on pasture contamination. The aim of the present study was to evaluate the anthelmintic activity of tanniniferous food from *Bauhinia pulchella* in goats and the potential impact on pasture contamination with the infective larval stage of gastrointestinal nematodes.

**Findings:**

Sixteen cross breed Boer goats that were naturally infected with gastrointestinal nematodes were fed tanniniferous concentrate from the leaves of *B. pulchella* and compared to a separate paddock of control animals without condensed tannin supplementation. A range of parasite characteristics were monitored throughout the 63 days of experimentation, including faecal egg count (FEC), egg hatching and relative numbers of hatched helminth larvae on herbage. Worm free tracer animals were used to assess the infective larval stage load of the contaminated pasture. The tanniniferous food did not reduce the combined FEC values, but egg hatching was significantly affected (*p* < 0.05). The pasture grazed by goats fed with tanniniferous food from *B. pulchella* showed reduced contamination through infective larval stages. Tracer goats maintained in paddocks grazed with animals fed with tanniniferous food had lower numbers of *Trichostrongylus colubriformis* than did those in the control group (86 % reduction).

**Conclusions:**

Condensed tannin from *B. pulchella* showed anthelmintic activity, affected egg viability and reduced pasture contamination, which led to the reduced infection of the animals by *T. colubriformis*.

## Findings

### Background

Condensed tannins (CT) are plant secondary metabolites that possess anthelmintic properties [1]. *Bauhinia pulchella* Benth is a leguminous plant native to Brazil where it is popularly known as mororó. This shrub, which ranges from 0.4 to 3.0 m in height, occurs in three phytogeographical regions: *Caatinga*, *Cerrado* and the Amazon [2]. *Bauhinia* species make up the diet of small ruminant livestock, particularly in *Caatinga* and *Cerrado* areas, where these plants are important sources of feed during the dry season [3]. The presence of CT indicates the potential of *Bauhinia* spp. as a nutraceutical option to control infections with gastrointestinal nematodes [4].

Previous research *in vitro* has shown the anthelmintic activity of CT monomers to inhibit egg hatch, larval exsheathment, development and association/penetration in the mucosae [1, 5, 6]. These effects disrupt the life cycle of the nematode and reduce the contamination of pasture with infective stage larvae. However, *in vivo* assays typically examine the anthelmintic activity of CT in female fecundity and/or adult worm burden [5, 6]. Thus, tannin-rich plants for controlling gastrointestinal nematodes are discounted, based upon failed *in vivo* trials without a complete analysis of the potential of these plants as an anthelmintic influencing alternative life cycle stages.

The aim of the present study was to evaluate the anthelmintic activity of a tanniniferous food from *B. pulchella* in goats and to determine the potential impact on pasture contamination with the infective larval stage of gastrointestinal nematodes.

### Methods

#### Plant materials

The leaves of *B. pulchella* were collected in Chapadinha City, Maranhão, Brazil. Botanists at the Herbarium of the Federal University of Bahia, Brazil identified the samples. The plant material was collected dried under shade, and ground. The fine powder of feed components (*B. pulchella,* pasture and hay) was subjected to the extraction process using aqueous acetone (70 %). The phenolic compounds, total phenol and tannin content were estimated according to Makkar et al. [7], Porter et al. [8], and Makkar et al. [9], respectively. Total phenol and total tannins were expressed as tannic acid equivalents, and CT was expressed as a leucocyanidin equivalent. Bromatological analyses were performed according to AOAC [10] and Van Soest [11].

#### Experimental animals

All procedures were approved through the Ethics Committee for the Animal Experimentation of the Federal University of Maranhão, Brazil under number 23115018061. Sixteen cross breed Boer mixed gender that were from 6 to 10 months of age, weighed 17.1 ± 2.7 kg, and had been dewormed at least 60 days prior to the experiment with Faecal Eggs Counts, (FEC) ≥ 400 were selected. The goats were distributed into two groups according to the FEC and live weight (LW) values. The control group received concentrate without CT (*n =* 8); and the treatment group received tanniniferous concentrate from the leaves of *B. pulchella* (180 mg of CT kg^−1^LW) (*n =* 8).

#### Experimental management

All concentrates provided to goats in this study, with or without *B. pulchella*, contained 14 % crude protein and 85 % total digestible nutrients. The amount of *B. pulchella* powder in the tanniniferous concentrate was determined according to the ratio of the CT quantified in the plant material and the CT concentration to be provided to the goats (180 mg of CT kg^−1^LW).

**Fig. 1:**
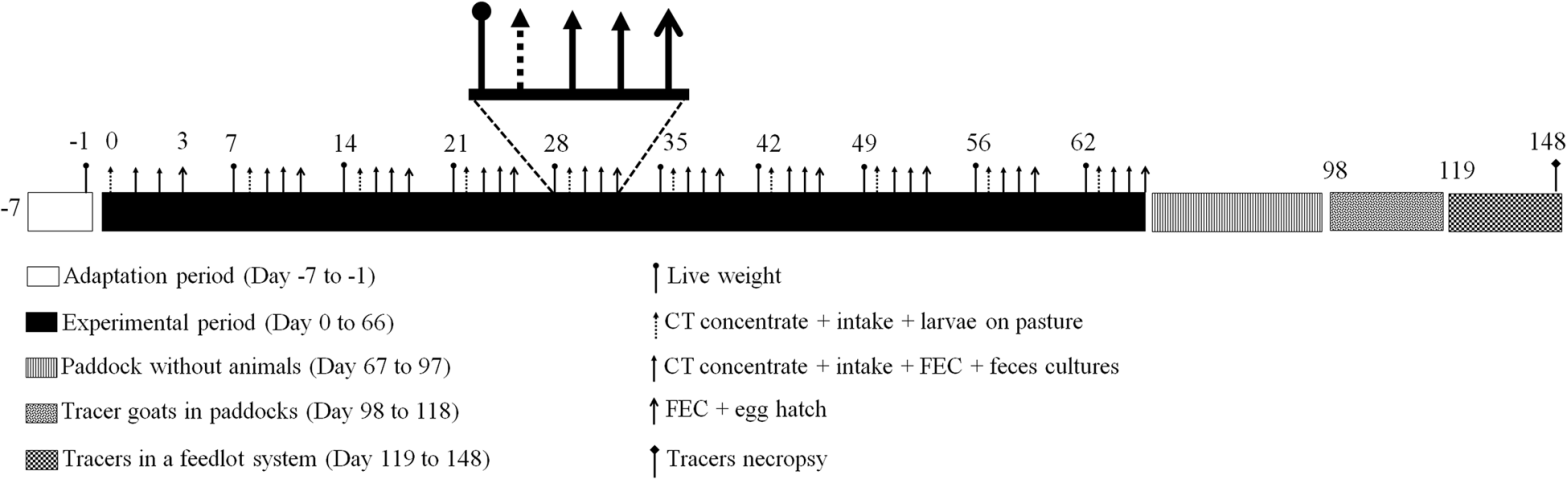
Schematic timeline of experimental procedures

The goats were subjected to experimental management during one week for adaptation. The experimental period was initiated after this week, and it lasted 66 days (Fig. 1). Prior to grazing all animals received a daily administration of the concentrate at 1.5 % of LW. The *B. pulchella* group received tanniniferous concentrate for three consecutive days per week. On the other days, this group consumed the same concentrate as did the control group. The goats were grazed 4 hours daily in two separate paddocks at a stocking density of eight goats ha^−1^. The pasture comprised *Panicum maximum* and was not grazed for at least 30 days. After grazing, the goats were housed in collection pens according to the experimental group and were provided ad libitum access to fresh water, mineral salt and Tifton hay (*Cynodon dactylon*). The goats were kept in individual pens and the concentrate offered for 2 hours. Subsequently, the individual refusals were weighed to calculate the feed intake percentage. The LW of the goats was analysed weekly (Fig. 1).

#### Parasitological analysis

The FEC was performed 3 consecutive days per week on the day following the provision of tanniniferous food from *B. pulchella* (Fig. 1). The FEC values were determined using the double centrifugation flotation test and represented as the average of these 3 days [12].

The eggs of gastrointestinal nematodes were purified weekly from pooled faecal samples obtained from each experimental group according to Bizimenyera et al. [13]. The eggs were incubated in quadruplicate at 27 °C for 24 h. The eggs and first stage larvae were counted, and the percent of hatching was calculated.

Faecal cultures were performed weekly for each group using the method according to Ueno and Gonçalves [14]. Infective stage larvae were identified according to VanWyk and Mayhew [15].

#### Pasture contamination

The number of infective stage larvae on herbage was analysed from the adaptation up to one month after the end of the experimental period (days 0 to 98) (Fig. 1). The herbage samples were collected once per week according to Taylor [16]. The extraction of infective stage larvae was performed after washing the herbage according to Niezen et al. [17]. The dry matter (DM) content of the herbage was determined. The herbage washings were passed through sieves (1 mm and 25 μm). The material on the 25-μm-mesh sieve was collected and centrifuged, and the number of infective stage larvae was determined. The results were expressed as the number of infective stage larvae per kilogram of dry matter (L_3_ kg^−1^ DM).

At day 98, two tracer worm-free animals grazed in each experimental paddock for 20 days. Subsequently, the tracers were maintained in a feedlot system, fed on Tifton hay for 1 month and necropsied after this period. The abomasum and intestinal nematodes were collected and identification performed as according to Ueno and Gonçalves [14]. The percentage efficacy (reduction) of the worm burden was calculated using Coles [18] formula.

#### Statistical analysis

Parasitological parameters (FEC values, egg hatch, and larval percentage from faeces cultures) and weight gain were compared between treatments, over the experimental days, using two-way ANOVA followed by Bonferroni post hoc test. The effect of the tanniniferous treatment duration on the egg hatching was analysed through linear regression. The Mann-Whitney *U* test was performed to analyse the effect of *B. pulchella* on the feed intake throughout the experimental period. Statistical procedures were performed using GraphPad Prism 6.0, consider *p* < 0.05.

### Results

#### Chemical and productive analysis

**Fig. 2:**
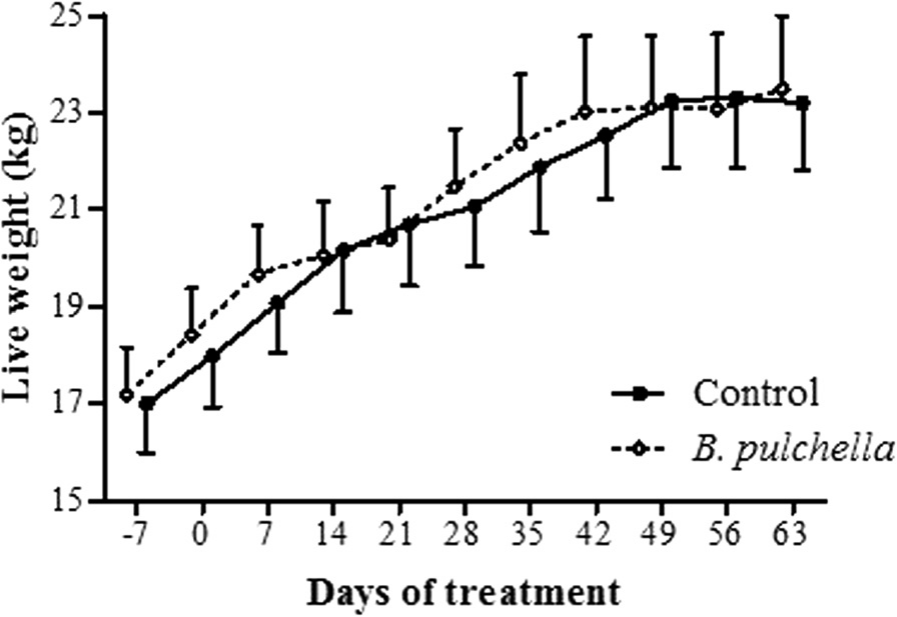
Live weight of experimental goats

The leaves of *B. pulchella* presented 13 % CT (Table 1). The concentrated intake of goats was not affected by the administration of tanniniferous food from *B. pulchella* (*p* > 0.05). The intake level was higher than 90 % in both experimental groups. The live weight was also similar between groups during the experiment (*p* > 0.05) (Fig. 2).

**Table 1:** Chemical composition (% of dry matter) of feed components

	*Bauhinia pulchella*	Pasture	Hay
Total phenols^a^	21.68	2.29	0.72
Total tannins^a^	15.44	1.53	0.41
Condensed tannins^b^	13.06	0.01	0.01
Crude protein	13.96	7.58	9.39
Ether extract	2.43	3.18	4.41
Neutral detergent fiber	59.03	79.46	75.90
Acid detergent fiber	44.48	44.91	44.90

^a^Total phenols and total tannins are expressed as tannic acid equivalent. ^b^Condensed tannins are expressed as the leucocyanidin equivalent

#### Parasitological analysis

**Fig. 3:**
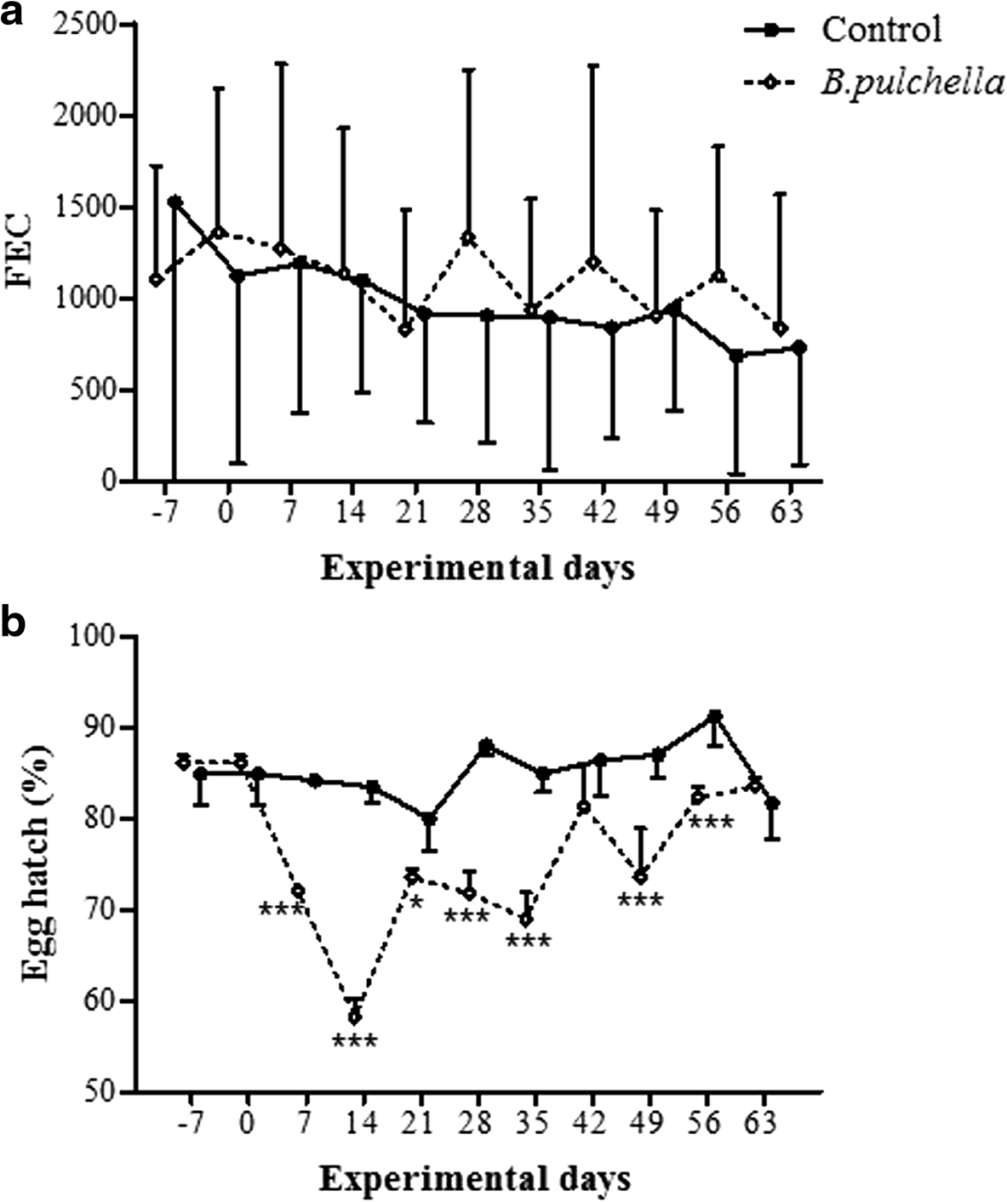
Faecal Eggs Count-FEC (**a**) and egg hatching (**b**) of the gastrointestinal nematodes infecting goats. The goats were provided concentrate without tannin (control group) and with tanniniferous food from *Bauhinia pulchella*. The FEC are reported as the mean values of three days of analysis. Significant difference: * *p* < 0.05; *** *p* < 0.001

The tanniniferous food from *B. pulchella* did not affect the FEC values (Fig. 3a). However, the egg hatch percentage in the treatment group was significantly lower than that in the control group in 7 out of 9 weeks assessed (days 7–35, 49 and 56) (Fig. 3b). The egg hatch percentage of the *B. pulchella* group gradually increased during the experiment (r^2^ = 0.69; F = 13.06; *p* = 0.01). The egg hatching was similar between the experimental groups on the last day of tanniniferous provision (day 63) (Fig. 3b).

**Fig. 4:**
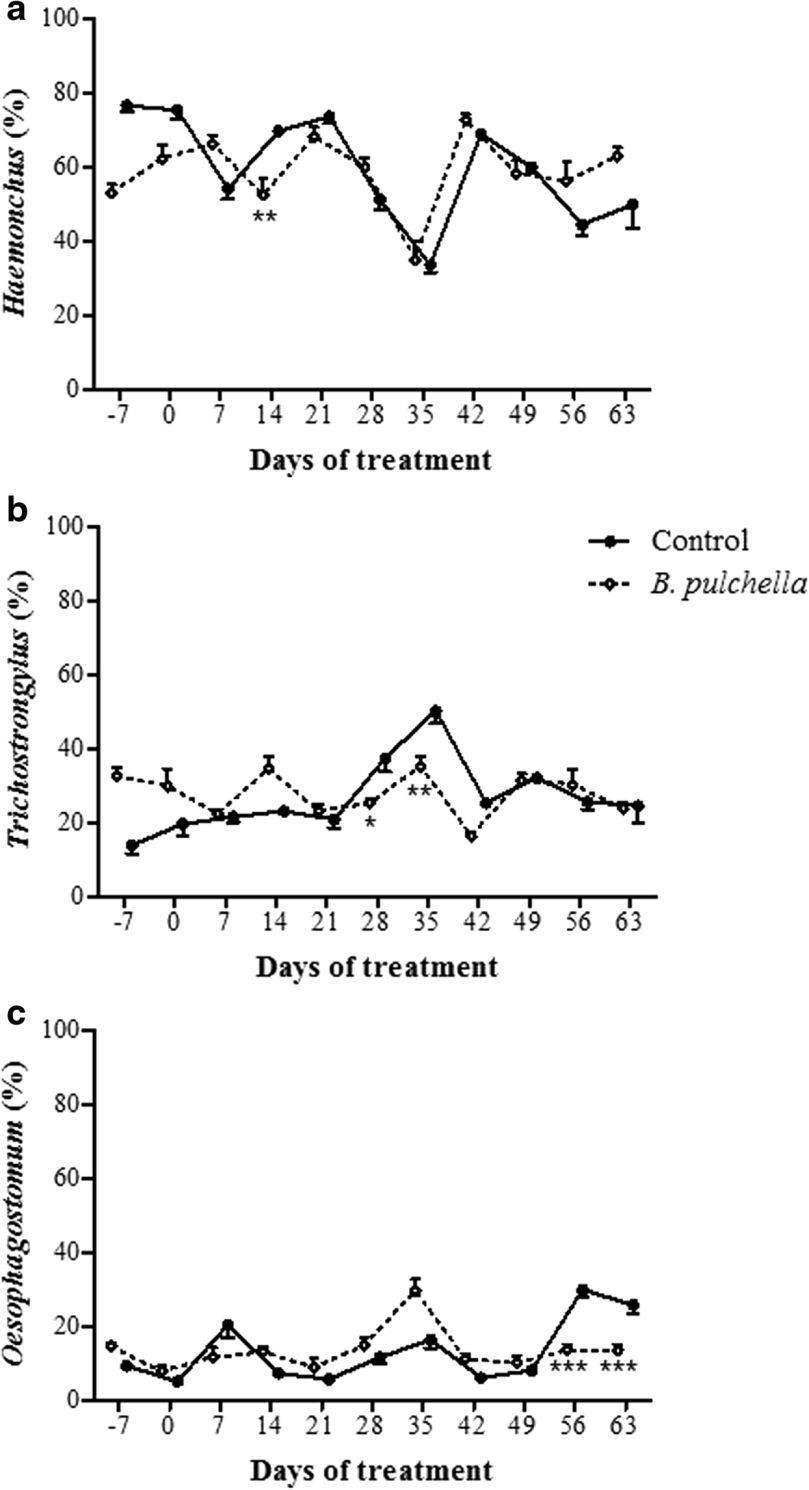
Infective stage larval frequency of gastrointestinal nematodes obtained from the faecal cultures from goats: (**a**) *Haemonchus*; (**b**) *Trichostrongylus*; and (**c**) *Oesohagostomum*. The goats were provided concentrate without tannin (control group) and with tanniniferous food from *Bauhinia pulchella*. The values are reported as the mean values of two days of analysis. Significant reduction compared with the control group: * *p* < 0.05; ** *p* < 0.01; *** *p* < 0.001

The goats were naturally infected with nematodes of the genus *Haemonchus*, *Trichostrongylus* and *Oesophagostomum*. The frequency of infective larval stage of these parasites ranged between the groups during the experimental period, however, they were sporadic and the overall trend showed no difference (Fig. 4).

#### Pasture contamination

**Fig. 5:**
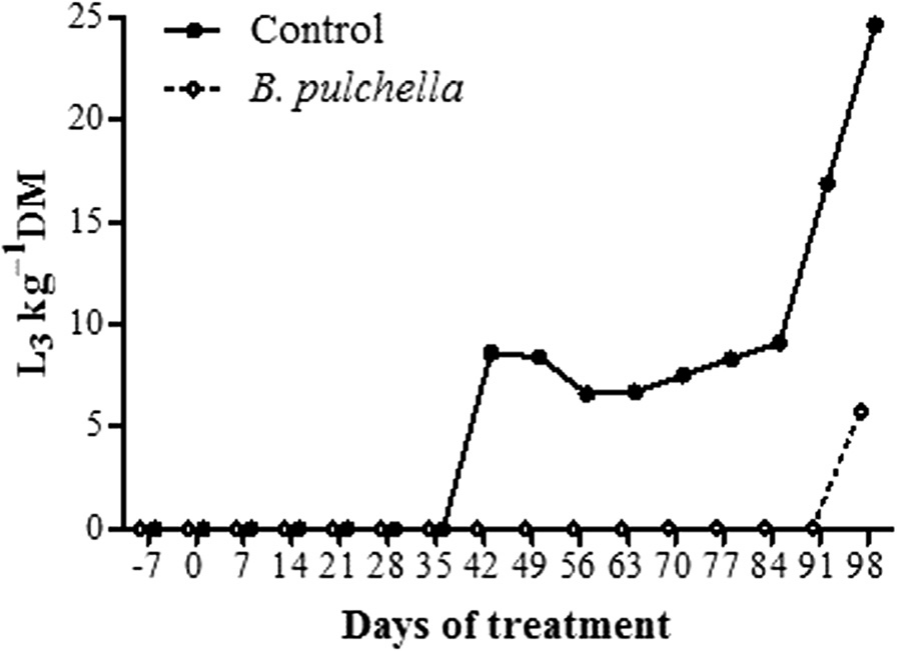
Infective stage larvae of gastrointestinal nematodes obtained from control and *Bauhinia pulchella* pasture group. The values are reported as number of larvae per kilo of dry matter

Infective stage larvae were identified from the control pasture after the 42nd experimental day (8.6 L_3_ kg^−1^ DM) (Fig. 5). *Haemonchus* and *Trichostrongylus* genus were the most prevalent genera. The maximum level of contamination in the control pasture was observed on the 98^th^ day (24.6 L_3_ kg^−1^ DM). The pasture grazed by goats fed with tanniniferous food from *B. pulchella* showed infective stage larvae only on the 98^th^ day: *Trichostrongylus* sp. contaminated the pasture at a rate of 5.8 L_3_ kg^−1^ DM (Fig. 5).

**Fig. 6:**
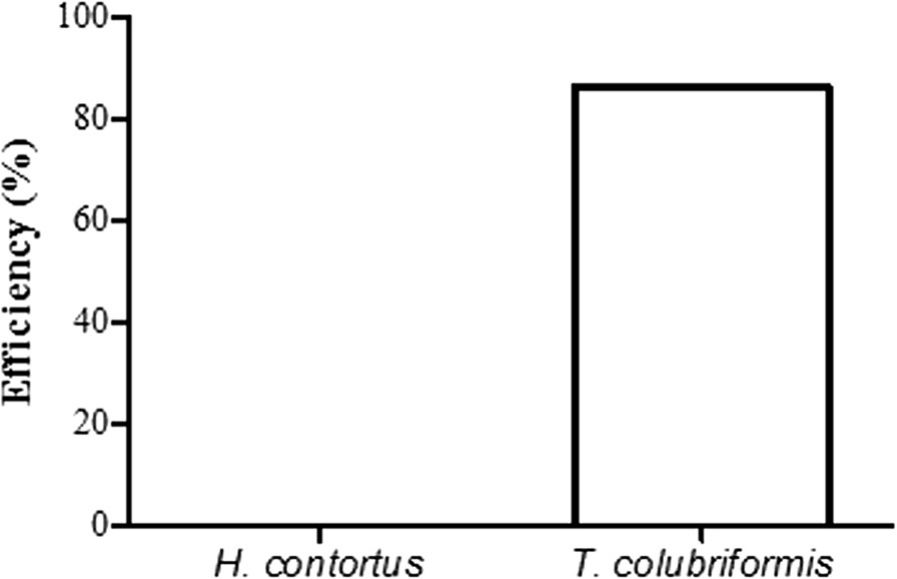
Reduction of *Haemonchus contortus* and *Trichostrongylus colubriformis* in tracer goats grazed in *Bauhinia pulchella* pasture group

The nematode burdens observed in tracer goats indicated the lower contamination level in the treatment compared with control paddock. Tracer goats maintained in the paddock grazed by animals fed with tanniniferous food from *B. pulchella* had lower numbers of *T. colubriformis* than those in the control group (reduction of 86 %) (Fig. 6). The number of *H. contortus* in tracer goats was not influenced by the tanniniferous provision from *B. pulchella*.

### Discussion

The percentage hatchability of eggs recovered from faeces of goats fed with *B. pulchella* CT was lower than that observed for the control group (Fig. 3b). This reduction in the availability might reflect the contact of the eggs with CT-free concentrate in the lumen or even in aggregates located around the female vulva [19]. CT binds to proteins that affect egg hatch by inhibiting changes in the egg shell permeability and enzymatic mechanisms or competition with hatching factor receptors on the egg shell [20]. However, the viability of eggs of gastrointestinal nematodes was temporarily affected through tanniniferous food from *B. pulchella*. The hatching percentage gradually increased and was similar in both groups at the end of the experimental period (Fig. 3b). Goats that consume tannin-rich plants developed physiological adaptations that inactivate these metabolites, such as the increased expression of salivary proteins [21]. The provision of tanniniferous food for three consecutive days per week could have gradually induced these adaptive processes, where the previous contact of the experimental goats with *B. pulchella* could have affected this time response. The concentration and frequency of *B. pulchella* provision and the anthelmintic efficacy should be studied further.

The tanniniferous food from *B. pulchella* decreased the pasture contamination with the infective larval stage of gastrointestinal nematodes. On the last day of analysis, the amount of larvae on the control pasture was 4.2 times higher than that on the pasture grazed by goats fed with tanniniferous food, 24.6 and 5.8 L_3_ kg^−1^ DM, respectively (Fig. 5). The tracer worm burdens indicate that the tanniniferous food decreased the pasture contamination with *T. colubriformis*, but not with *H. contortus* (Fig. 6). This decrease in the pasture contamination with *T. colubriformis* could be associated with a break in the parasite cycle due to reduced egg hatch.

As fecundity of female worms can differ between species of gastrointestinal nematodes, the number of eggs for hatching and infective larval stage on the pasture might differ according to the parasite species affected with tanniniferous food [22]. Contact with tannin-rich plants induces adaptations not only in the host but also in the parasite, which makes these organisms less sensitive to these metabolites [23]. The anthelmintic effect of CT from *B. pulchella* is the inhibition of egg hatch, which suggests that this life-cycle stage is subjected to higher selection pressure to develop adaptation mechanisms in response to tannins. Tracer worm burdens indicate that the tanniniferous food decreased the pasture contamination with *T. colubriformis*, but not with *H. contortus*.

The presence of CT in the ruminant diet can cause toxic and anti-nutritional effects or improve the performance of the host [24]. In the present study, the concentrate containing 180 mg of CT kg^−1^ LW from *B. pulchella* did not affect the weight gain and feed intake of the goats. These results indicate that this concentration of CT from *B. pulchella* is not anti-nutritional, and its provision did not show acute toxicity when provided as a concentrate. Previous contact with *B. pulchella* might have induced physiological adaptations on goats [25], thus enabling the intake of the tanniniferous food without affecting nutritional and productive aspects. The CT from *B. pulchella* have anthelmintic activity, through reduced the egg viability and subsequent reduced pasture contamination, which leads to the reduced infection of the animals with *T. colubriformis*, but not with *H. contortus*.
